# FATHMM-XF: accurate prediction of pathogenic point mutations via extended features

**DOI:** 10.1093/bioinformatics/btx536

**Published:** 2017-09-05

**Authors:** Mark F Rogers, Hashem A Shihab, Matthew Mort, David N Cooper, Tom R Gaunt, Colin Campbell

**Affiliations:** 1 Intelligent Systems Laboratory, University of Bristol, Bristol, UK; 2 MRC Integrative Epidemiology Unit (IEU), University of Bristol, Bristol, UK; 3 Institute of Medical Genetics, Cardiff University, Cardiff, UK

## Abstract

**Summary:**

We present *FATHMM-XF*, a method for predicting pathogenic point mutations in the human genome. Drawing on an extensive feature set, *FATHMM-XF* outperforms competitors on benchmark tests, particularly in non-coding regions where the majority of pathogenic mutations are likely to be found.

**Availability and implementation:**

The *FATHMM-XF* web server is available at http://fathmm.biocompute.org.uk/fathmm-xf/, and as tracks on the Genome Tolerance Browser: http://gtb.biocompute.org.uk. Predictions are provided for human genome version GRCh37/hg19. The data used for this project can be downloaded from: http://fathmm.biocompute.org.uk/fathmm-xf/

**Supplementary information:**

Supplementary data are available at *Bioinformatics* online.

## 1 Introduction

Many classifiers have been proposed for predicting the impact of single-nucleotide variants (SNVs) in the human genome (see Liu *et al.*, 2017). Initially these focused on non-synonymous mutations in coding regions of the genome, but most documented pathogenic SNVs come from non-coding regions, so more recent methods make predictions genome wide (Kircher *et al.*, 2014; Shihab *et al.*, 2015). CADD (Kircher *et al.*, 2014) has emerged as a standard for predicting pathogenic SNVs, although its performance has been challenged (Liu *et al.*, 2017). The recent GAVIN method adjusts CADD scores in a gene-specific manner, achieving greater accuracy than CADD, whilst assigning distinct *Pathogenic* and *Benign* labels that simplify interpretation (van der Velde *et al.*, 2017).

Here we present FATHMM with an eXtended Feature set (*FATHMM-XF*) which yields highly accurate predictions for SNVs across the entire human genome. *FATHMM-XF* assigns a confidence score (a *p*-score) to every prediction, to simplify interpretation, and focus analysis on a subset of high-confidence predictions (*cautious classification*). In all tests, *FATHMM-XF* matches or outperforms competing methods, with its best performance in non-coding regions, where the majority of pathogenic SNVs are likely to be found. With cautious classification, *FATHMM-XF* consistently exceeds 94% accuracy on subsets of 80% of the highest-confidence predictions from benchmark test sets.

## 2 Materials and methods

To build *FATHMM-XF* we use supervised machine learning with labeled examples ascribed to pathogenic (positive) or benign (neutral) mutations. We obtain positive examples from the Human Gene Mutation Database (Stenson *et al.*, 2017) (HGMD), and neutral examples from the 1000 Genomes Project (The 1000 Genomes Project Consortium, 2012). We restrict neutral data to SNVs with a global minor allele frequency ≤1% and remove any that appear in the pathogenic dataset. To mitigate potential bias, we filter neutral examples, selecting only those within 1000 positions of a pathogenic mutation (Supplementary Section S2). In addition, we remove sex chromosomes X and Y to avoid potential biases that might arise when allosomes are included. Our final training set consists of 156 775 coding examples and 25 720 non-coding. We characterise SNVs using features from 27 data sets (herein called *feature groups*) from ENCODE (The ENCODE Project Consortium, 2012) and NIH Roadmap Epigenomics (Bernstein *et al.*, 2010) that have proved informative in other domains (Shihab *et al.*, 2015, 2017b). We construct four additional feature groups from conservation scores, the Variant Effect Predictor (McLaren *et al.*, 2016); annotated gene models, and the DNA sequence itself (Supplementary Section S3). We convert feature groups into kernels to evaluate different combinations and kernel-based models. *k*-fold cross-validation is commonly used to evaluate models, but can introduce bias if, for example, the same gene is represented in both training and test sets. Instead, we use leave-one-chromosome-out cross-validation (LOCO-CV): for each fold we set aside one chromosome for testing and use the remaining chromosomes for training.

We use Platt scaling (Platt, 1999) to assign a *p*-score to each prediction (the probability that a particular SNV is pathogenic). For cautious classification, we then establish confidence thresholds to analyse sub-populations of high-confidence predictions.

## 3 Results

For non-coding regions, the best model incorporates five feature groups, achieving 92.3% accuracy in LOCO-CV (Supplementary Table S6). Briefly, these feature groups encapsulate sequence conservation, proximity to genomic features (e.g. splice sites or transcription start sites) and chromatin accessibility. Cautious classification reaches 99% peak accuracy at a *p*-score threshold of τ=0.96 (Supplementary Fig. S2). This high-confidence subset of examples (*p* ≥ 0.96 or ≤ 0.04) comprises nearly 40% of test examples, demonstrating that the threshold is not prohibitively restrictive. Relaxing the threshold enlarges this subset dramatically whilst retaining high accuracy: at τ=0.80, we cover 90% of examples with accuracy over 95% (Supplementary Section S4).

For coding regions, the best model uses six feature groups, reaching 88.0% accuracy (Supplementary Table S8). Again, conservation features are most informative, along with proximity to genomic features and nucleotide sequence features (Supplementary Section S3). Cautious classification achieves peak accuracy of 98% at τ=0.97 (Supplementary Fig. S2). This highest-confidence subset again comprises nearly 40% of examples; at τ=0.80, it includes 80% of examples with accuracy above 94.0%. We use these peak accuracy thresholds (0.96 for non-coding, 0.97 for coding) in subsequent analyses.

We compared *FATHMM-XF* with four genome-wide SNV prediction methods: CADD (Kircher *et al.*, 2014), DANN (Quang *et al.*, 2014), FATHMM-MKL (Shihab *et al.*, 2015) and GAVIN (van der Velde *et al.*, 2017). When we compared FATHMM-MKL LOCO-CV test results with competitors evaluated on the same data, *FATHMM-XF* achieved the highest accuracy of all, at 93% (Supplementary Section S5). In coding regions, *FATHMM-XF* and its closest competitor, GAVIN, yielded similar accuracy (88 and 89%, respectively). As reported earlier, *FATHMM-XF* yielded exceptionally high accuracy in cautious classification on these data, whilst consistently yielding predictions for nearly 40% of examples.

To evaluate how well *FATHMM-XF* will generalise, we tested all methods on test sets we assembled from ClinVar data (Landrum *et al.*, 2014) (Supplementary Section S5). After removing any ClinVar examples found in our training sets, the test sets comprised 31 099 non-coding and 62 884 coding SNVs. In non-coding regions, *FATHMM-XF* matches or outperforms other methods, reaching 89% accuracy and 0.97 area under the ROC curve (AUC, Table 1, top). FATHMM-MKL yields comparable accuracy, but tends to under-perform the new model. GAVIN achieves higher MCC and PPV scores at the expense of lower accuracy. In cautious classification, *FATHMM-XF* yields exceptionally high scores, covering 30.9% of examples. In coding regions, it reaches 88% accuracy and 0.96 AUC (Table 1, bottom). GAVIN yields nominally higher accuracy (and, notably, 26% higher than CADD, upon which it is based), but at lower MCC and PPV. With cautious classification, *FATHMM-XF* again yields exceptional performance, covering 42.4% of examples. *FATHMM-XF* at its default threshold covers 100% of test examples, as do the other methods tested.

**Table 1.** FATHMM-XF yields state-of-the-art accuracy on unseen ClinVar examples in both non-coding regions and coding regions.

Non-coding regions						
Method	Acc.	AUC	Sens.	Spec.	MCC	PPV
FATHMM-XF	0.89	0.97	0.95	0.84	0.53	0.36
* Cautious* (τ=0.96)	*0.96*	*0.99*	*0.99*	*0.93*	*0.87*	*0.82*
FATHMM-MKL	0.88	0.95	0.94	0.82	0.49	0.33
GAVIN	0.87	—	0.82	0.93	0.61	0.52
CADD (v1.3)	0.64	0.95	0.98	0.30	0.18	0.12
DANN	0.61	0.95	0.99	0.23	0.15	0.11

Coding regions	Acc.	AUC	Sens.	Spec.	MCC	PPV

FATHMM-XF	0.88	0.96	0.84	0.92	0.76	0.83
* Cautious* (τ=0.97)	*0.97*	*0.99*	*0.94*	*1.00*	*0.96*	*0.99*
GAVIN	0.89	—	0.90	0.87	0.74	0.76
FATHMM-MKL	0.80	0.90	0.91	0.70	0.56	0.58
CADD (v1.3)	0.63	0.91	0.98	0.29	0.30	0.38
DANN	0.60	0.89	0.99	0.20	0.25	0.36

*Note*: (*Top*) FATHMM-XF yields the highest accuracy on unseen ClinVar examples for non-coding regions, outperforming its nearest competitor, FATHMM-MKL. Cautious classification yields exceptionally high scores, yielding predictions for 31% of examples. (*Bottom*) FATHMM-XF yields higher accuracy, AUC, MCC and PPV scores than competitors on unseen ClinVar examples in coding regions. The lone exception is GAVIN, with nominally higher accuracy. Cautious classification again achieves extremely high scores, yielding predictions for more than 42% of test examples.

## 4 Discussion

At default thresholds, *FATHMM-XF* matches or outperforms competing methods using an eclectic mixture of data sources. Even when all methods are optimised, *FATHMM-XF* yields substantially higher accuracy in all of our tests (Supplementary Figs S7–S10). Under cautious classification, accuracy exceeds 95%, whilst producing predictions for up to 80% of positions genome-wide. While the proposed classifiers achieve high accuracy, further improvement seems possible. Notably, all methods exhibit low PPV on non-coding data except for *FATHMM-XF’s* cautious classification. Analysis of these variants (Supplementary Fig. S1) reveals differences in the proportions of intron and UTR variants represented in the training and test sets. Hence region-specific models may improve performance in non-coding regions, just as GAVIN’s gene-specific thresholding improves accuracy for CADD scores—by up to 26 percentage points in our tests. We will explore these approaches in future work. The *FATHMM-XF* web server for GRCh37/hg19 is available at fathmm.biocompute.org.uk/fathmm-xf, and as tracks on the Genome Tolerance Browser (gtb.biocompute.org.uk; Shihab *et al.*, 2017a).

## Funding

MR was supported by the Engineering and Physical Sciences Research Council (EPSRC) grants [EP/M01715X/1] and [EP/K008250/1]. TRG was supported by Medical Research Council Integrative Epidemiology Unit (MRC IEU) [MC UU 12013/8]. MM & DNC gratefully acknowledge the financial support of Qiagen Inc. through a licence agreement with Cardiff University.

*Conflict of Interest*: none declared.

## Supplementary Material

Supplementary Data

## References

[btx536-B1] Bernstein B.E. et al (2010) The NIH roadmap epigenomics mapping consortium. Nat. Biotechnol., 28, 1045–1048. 20944595 10.1038/nbt1010-1045 PMC3607281

[btx536-B2] Kircher M. et al (2014) A general framework for estimating the relative pathogenicity of human genetic variants. Nat. Genet., 46, 310–315. 24487276 10.1038/ng.2892 PMC3992975

[btx536-B3] Landrum M.J. et al (2014) ClinVar: public archive of relationships among sequence variation and human phenotype. Nucleic Acids Res., 42, D980–D985. 24234437 10.1093/nar/gkt1113 PMC3965032

[btx536-B4] Liu X. et al (2017) The performance of deleteriousness prediction scores for rare non-protein-changing single nucleotide variants in human genes. J. Med. Genet., 54, 134–144. 27999115 10.1136/jmedgenet-2016-104369 PMC5736365

[btx536-B5] McLaren W. et al (2016) The ENSEMBL variant effect predictor. Genome Biol., 17, 122. 27268795 10.1186/s13059-016-0974-4 PMC4893825

[btx536-B6] Platt J. (1999) Probabilistic outputs for support vector machines and comparison to regularised likelihood methods. In: *Advances in Large Margin Classifiers*, MIT Press, Cambridge, MA, pp. 61–74.

[btx536-B7] Quang D. et al (2015) DANN: a deep learning approach for annotating the pathogenicity of genetic variants. Bioinformatics, 31, 761–763. 25338716 10.1093/bioinformatics/btu703 PMC4341060

[btx536-B8] Shihab H.A. et al (2015) An integrative approach to predicting the functional effects of non-coding and coding sequence variation. Bioinformatics, 31, 1536–1543. 25583119 10.1093/bioinformatics/btv009 PMC4426838

[btx536-B9] Shihab H.A. et al (2017a) GTB—an online genome tolerance browser. BMC Bioinformatics, 18, 20. 28061747 10.1186/s12859-016-1436-4 PMC5219737

[btx536-B10] Shihab H.A. et al (2017b) Hipred: an integrative approach to predicting haploinsufficient genes. Bioinformatics, 33, 1751–1757. 28137713 10.1093/bioinformatics/btx028 PMC5581952

[btx536-B11] Stenson P.D. et al (2017) The human gene mutation database: towards a comprehensive repository of inherited mutation data for medical research, genetic diagnosis and next-generation sequencing studies. Hum. Genet., 136, 665–677. 28349240 10.1007/s00439-017-1779-6 PMC5429360

[btx536-B12] The 1000 Genomes Project Consortium. (2012) An integrated map of genetic variation from 1, 092 human genomes. Nature, 491, 56–65. 23128226 10.1038/nature11632 PMC3498066

[btx536-B13] The ENCODE Project Consortium. (2012) An integrated encyclopedia of DNA elements in the human genome. Nature, 489, 57–74. 22955616 10.1038/nature11247 PMC3439153

[btx536-B14] van der Velde K.J. et al (2017) Gavin: Gene-aware variant interpretation for medical sequencing. Genome Biol., 18, 6. 28093075 10.1186/s13059-016-1141-7 PMC5240400

