# Supplementary material for: FATHMM-XF: accurate prediction of pathogenic point mutations via extended features
Source: Bioinformatics. 2017 Sep 5;34(3):511–3. doi: 10.1093/bioinformatics/btx536 (PMC5860356; doi:10.1093/bioinformatics/btx536)
Supplement: Supplementary Data [file btx536_supplementary.pdf]

Genome analysis

Supplementary information for FATHMM-XF: accurate prediction of pathogenic point mutations via extended features

Mark F. Rogers [1,*], Hashem A. Shihab [2], Matthew Mort [3], David N. Cooper [3], Tom R. Gaunt [2] and Colin Campbell [1,*]

[1] Intelligent Systems Laboratory, University of Bristol, Bristol, BS8 1UB, UK.
[2] MRC Integrative Epidemiology Unit (IEU), University of Bristol, Bristol, BS8 2BN, UK.
[3] Institute of Medical Genetics, Cardiff University, Cardiff, CF14 4XN, UK.

* To whom correspondence should be addressed.

1 Leave-one-chromosome-out cross-validation (LOCO-CV)

We evaluated all models using LOCO-CV. For each fold we leave out one test chromosome and use the remaining chromosomes to train the model, applying the same model parameters for all folds. Except where noted, we trained models using balanced sets of 1,000 positive and 1,000 negative examples. These relatively small training subsets yield accuracy nearly as high as the final model but take less time to train, and this approach allowed us to estimate variability that arises from using randomised training sets. For testing we can use all available examples for the left-out chromosome, resulting in unbalanced test sets in most cases. For this reason we report balanced accuracy for all tests.

2 Labeled examples

We constructed our pathogenic (positive) dataset using somatic point mutations from the HGMD database (Stenson *et al.*, 2017) while we used SNVs from the 1,000 Genomes Project (The 1000 Genomes Project Consortium, 2012) as neutral examples. Bias may be introduced if benign mutations are located in genomic regions that differ substantially from regions containing pathogenic mutations, such as examples from different genes. To ensure the locations of putative benign mutations approximate those of pathogenic mutations, we select only those putative benign mutations found within a 1000-nt window of some pathogenic mutation. This issue has been evaluated more thoroughly in previous studies (Ritchie *et al.*, 2014; Kircher *et al.*, 2014; Shihab *et al.*, 2015). In addition, we restrict our data to the autosomes (Table 1), as allosomes (sex chromosomes X and Y) have been shown to evolve at a different rate than the autosomes (see, e.g., (Charlesworth *et al.*, 1987)) and hence may have different sequence conservation profiles. As a result, we are wary of models or predictions that do not consider this distinction.

We used the Variant Effect Predictor (McLaren *et al.*, 2016), to assess the distribution of examples in our training data and compared them with our ClinVar test sets (Supplementary Figures 1 and 2). In coding regions, we found that the majority of pathogenic examples are missense mutations, causing a change in the resulting amino acid, whilst the neutral examples are balanced between missense and synonymous mutations. In non-coding regions, a large proportion of pathogenic examples reside near splice sites, whilst neutral examples tend to be spread more evenly across introns. It is important to note that the overwhelming majority of human genes undergo alternative splicing, hence mutations tend to fall within multiple transcripts, hence receive multiple annotations. As a result, the proportions shown in these graphs do not sum to 1. In addition, many examples in coding regions may also function as non-coding (*Noncoding alternatives*), for example, when they fall within a cassette exon that is omitted from some transcripts.

3 Feature groups and kernels

Subsequent to our previous work on *FATHMM-MKL*, we obtained a variety of additional data sets from ENCODE (The ENCODE Project Consortium, 2012) and from NIH Roadmap Epigenomics (Bernstein *et al.*, 2010). These resources provided us with nearly 30 data sources that have proved informative in predicting haploinsufficient genes, for example (Shihab *et al.*, 2017b). Recently we have also derived new features from the Variant Effect Predictor (McLaren *et al.*, 2016), from annotated gene models, and from patterns in nucleotide sequence. We converted each of these feature groups into a set of kernels consisting of: two conservation-based kernels; a nucleotide spectrum kernel; two kernels designed to encapsulate the genomic context (gene region, amino acid changes, and proximity to gene features), and 27 kernels based on ENCODE data.

© The Author 2015. Published by Oxford University Press. All rights reserved. For permissions, please e-mail: journals.permissions@oup.com

Chromosome	Coding		Non-coding	
	1000G	HGMD	1000G	HGMD
1	11,240	5,484	1,909	590
2	7,100	3,674	1,510	611
3	5,540	3,503	909	445
4	4,236	1,450	932	231
5	4,826	1,637	939	337
6	6,358	1,715	1,109	237
7	5,096	3,286	872	406
8	3,609	1,136	712	251
9	4,478	1,697	976	292
10	4,164	1,449	719	180
11	7,099	4,382	1,243	596
12	5,344	2,962	920	371
13	1,682	1,384	376	257
14	4,016	1,639	481	121
15	3,416	1,940	695	281
16	4,984	2,504	1,083	340
17	6,208	3,255	1,008	595
18	1,700	843	500	121
19	9,173	3,131	860	230
20	2,875	953	381	99
21	1,403	591	370	57
22	2,814	799	440	128
Total	107,361	49,414	18,944	6,776

Table 1. **Distributions of training examples by chromosome** shows the number of examples available for testing and training in LOCO cross-validation. These data are unbalanced, with up to 3.7 times as many neutral (1000G) examples as positive (HGMD) examples in coding regions and up to 6 times as many in non-coding regions. Note that for FATHMM-XF, only autosomal chromosomes were used in training and testing the method.

3.1 Conservation and ENCODE data

As in previous studies (Shihab *et al.*, 2015; Rogers *et al.*, 2015; Shihab *et al.*, 2017b) we evaluated distinct ENCODE datasets as potential feature groups for these classifiers. Broadly speaking, we divide these datasets into eight categories:

- *Genomic and Evolutionary*: where appropriate, we used a number of genomic properties such as gene length, number of transcripts and the average number of predicted protein domains across transcripts. In addition, we used a comprehensive set of conservation-based measures, such as dN/dS ratios between human and 65 different species (one-to-one orthologues). We also used several conservation based measures, e.g., PhyloP (Pollard *et al.*, 2010) and PhastCons (Siepel *et al.*, 2005) scores, derived from the multiple sequence alignment of 46 and 100 vertebrate genomes to the human genome (Blanchette *et al.*, 2004).
- *Histone Modifications*: we used ChIP-Seq peak calls for 14 histone modifications across 45 cell lines from ENCODE (The ENCODE Project Consortium, 2012) and narrow, broad and gapped regions of enrichment based on consolidated epigenomes from the NIH Roadmap project (Kundaje *et al.*, 2015).
- *Open Chromatin*: we used DNase-Seq and Formaldehyde-Assisted Isolation of Regulatory Elements (FAIRE) peak calls across 119 cell lines from ENCODE and narrow, broad and gapped regions of enrichment based on consolidated epigenomes from the NIH Roadmap project.
- *Transcription Factor Binding Sites*: based on PeakSeq and SPP peak calls for 119 transcription factors across 77 cell lines from ENCODE.
- *Gene Expression*: based on RNA-seq signal coverage using consolidated epigenomes from NIH Roadmap Epigenomics.
- *Methylation*: based on whole genome bisulphite sequencing (WGBS) from NIH Roadmap Epigenomics.
- *Digital Genomic Footprinting Sites*: for transcription factor recognition sequences within DNase-hypersensitive sites using consolidated epigenomes from the NIH Roadmap Epigenomics Project.
- *Networks*: we used measures of centrality from cell-type specific interactome and tissue-specific co-expression networks.

3.2 Sequence features

One goal for our models is to learn the sequence characteristics that are most susceptible to pathogenic mutations in both coding and non-coding regions. As a simple method for capturing the disruption that may occur in the sequence surrounding a mutation, we use *spectrum* kernels (Leslie *et al.*, 2002) to compare the composition of k-mers within a region before and after a mutation is applied to a sequence. Given a mutation and its flanking sequence, we obtain the k-spectra for the wild-type and mutated versions of the sequence and concatenate these features to provide a picture of the region before and after mutation. Formally, borrowing notation from (Leslie *et al.*, 2002), if the k-spectrum of an input sequence s is the set of all k-length contiguous subsequences, then we define a feature map of all possible subsequences a of length k from alphabet $\mathcal{A}$ as follows:

$$\Phi_k(s) = (\phi_a(s))_{a \in \mathcal{A}^k} \tag{1}$$

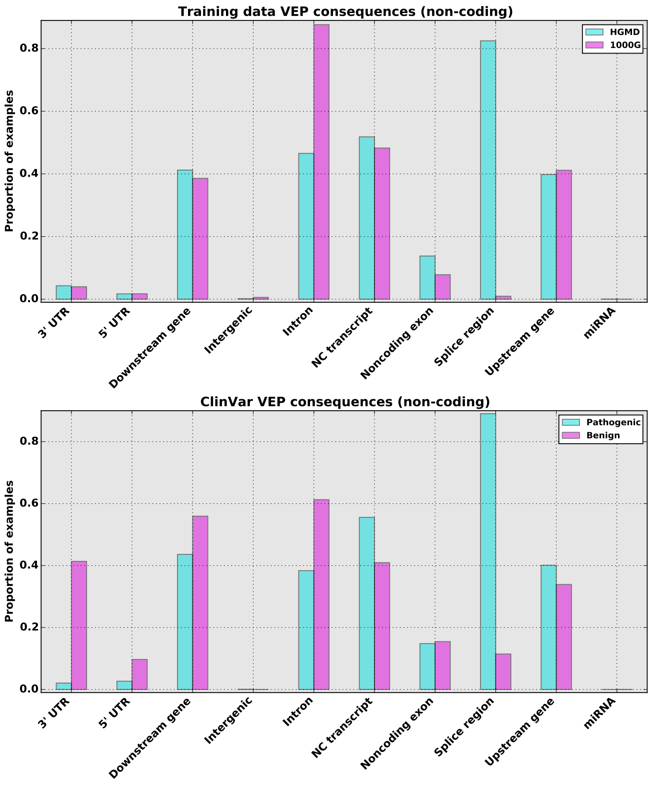

Fig. 1. Distributions of consequence annotations from the Variant Effect Predictor highlight differences between pathogenic and neutral examples in non-coding regions within the *FATHMM-XF* training data (top) and the ClinVar benchmark (bottom). In both data sets, splicing regions are by far the most prevalent group within positive (Pathogenic/HGMD) examples. In the *FATHMM-XF* training set, intronic variants are prominent amongst the neutral (1000G) data, whilst the ClinVar neutral (Benign) data also feature UTRs and locations downstream of genes.

where $\phi_a(s)$ is the count of the number of times sequence a occurs in sequence s, from which a kernel matrix can be readily derived (Campbell and Ying, 2011). We found that these features perform competitively on their own, and improve prediction accuracy in our merged-kernel tests. As this approach makes no assumptions about the kind of RNA binding proteins that may be impacted by a particular mutation, it may obviate the need to find and assess known motifs.

For these features we optimise two relevant parameters: the size of the window w flanking each mutation, and the maximum k-mer size, k. For a single-point mutation we expect the disruption to be confined to a relatively small region around the mutation. This restricts the useful window size and in turn, the maximum k-mer sizes that will be relevant. For both coding and non-coding models we performed a grid search over these sizes, for $k \in [1, \ldots, 5]$ and $w \in [1, \ldots, 10]$.

3.3 Genomic context features

We also include features that describe the genomic context where a mutation occurs. For coding regions we base these features on information from the ENSEMBL Variant Effect Predictor (VEP). The VEP provides characteristics for specific genomic locations that we can exploit to predict the likely impact of a SNV. These may include transcript features and amino acids impacted by a mutation, relative allele frequencies, and scores from pathogenic variant predictors such as SIFT and PolyPhen (Adzhubei *et al.*, 2010). To mitigate potential bias we are careful not to include these other scores, nor do we include features such as PubMed IDs that may have been used to curate SNV databases. Hence our features include only the following elements:

- *Consequence:* the VEP provides these as annotations of 35 types of changes to associated transcripts, such as *3' UTR variant*, *missense variant* or *TF binding site variant*. We represent these using 35-element binary vectors (one bit per annotation), plus a count of the number of transcripts possibly impacted. Note that we do not encode the impact levels provided on the VEP website (HIGH, MODERATE, MODIFIER, LOW); instead we allow our model to learn priorities in training.
- *Amino acid:* the amino acids inferred by the reference and allele nucleotides. To capture the change in amino acid composition, we construct two sets of features: two 20-element binary vectors that reflect the reference and allele residues, respectively, and two real-valued vectors that represent specific residue characteristics: molecular weight, hydrophobicity, occurrence frequency, dissociation constants for the $COOH$ and NH_3^+ groups, and the pH at the isoelectric point.

We apply the VEP features only to our coding predictor, as amino acid features are not relevant to SNVs in non-coding regions, and there are far more non-coding positions than coding positions: such a vast number of VEP queries would be impractical for creating a genome-wide database.

We concatenate two vectors to encapsulate all of the VEP features used in the model for coding regions. The *Consequences* features consist of a transcript count (the number of transcripts that may be impacted by a mutation) and a vector of binary flags that represent the possible consequences

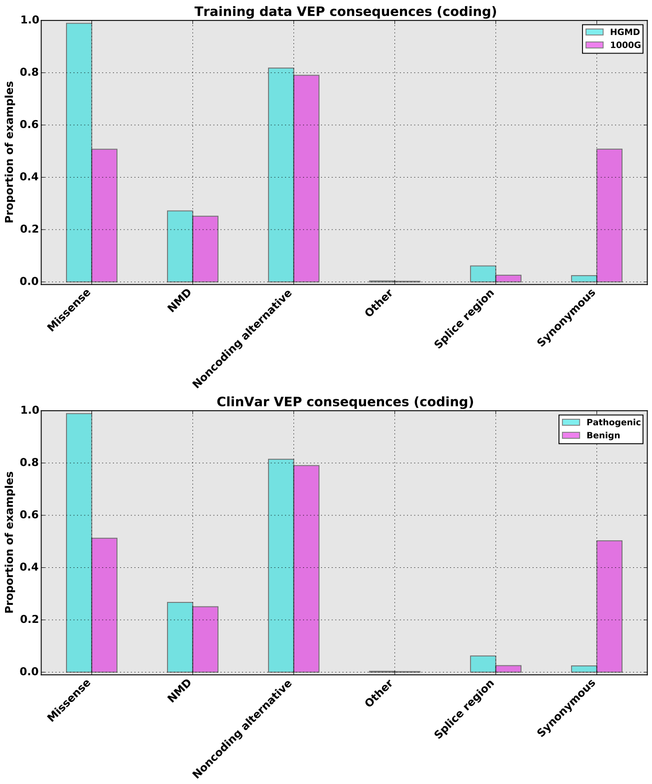

Fig. 2. The distributions of consequence annotations from the Variant Effect Predictor highlight differences between pathogenic and neutral examples in coding regions within the *FATHMM-XF* training data (top) and the ClinVar benchmark (bottom). Here we see that both datasets have nearly identical distributions of features.

returned by the VEP (Table 2). The *Amino acids* features consist of two vectors that encapsulate the possible amino acids associated with each mutation. The first amino acid vector represents the amino acids associated with the wild-type sequence, and the second represents the amino acids associated with the mutation. Instead of using a simple binary flag, we use counts to record the number of transcripts impacted by each amino acid change. The accuracy of this model is recorded as *Distance* in Table 4.

transcript ablation	*splice region variant*	*start lost*	*5 prime UTR variant*
upstream gene variant	*splice acceptor variant*	*regulatory region amplification*	*transcript amplification*
incomplete terminal codon variant	*downstream gene variant*	*3 prime UTR variant*	*feature elongation*
splice donor variant	*stop retained variant*	*inframe insertion*	*non coding transcript exon variant*
TFBS ablation	*stop gained*	*regulatory region variant*	*inframe deletion*
synonymous variant	*TFBS amplification*	*intron variant*	*feature truncation*
frameshift variant	*coding sequence variant*	*missense variant*	*NMD transcript variant*
TF binding site variant	*stop lost*	*intergenic variant*	*protein altering variant*
mature miRNA variant	*regulatory region ablation*	*non coding transcript variant*	

Table 2. **Consequence codes encapsulated in the VEP features.** The coding region classifier uses a binary vector to identify which of these consequences is annotated for a particular mutation.

3.4 Distance features

For non-coding SNVs we employ a related, but simpler approach: we measure the distance from each SNV to gene features annotated in the ENSEMBL gene models: *start codon*, *stop codon*, *gene*, *UTR*, *CDS* and *exon*. This approach is simple, yet should enable our models to learn relationships between SNVs and important gene elements. For example, exon boundaries help to identify mutations close to splice sites. Similarly, 5' gene boundaries identify mutations close to transcription start sites or promoter regions. To capture this information, we establish a window w around each mutation and measure the distance to the nearest example of each element. The features are then the distances to six element types, mapped onto the range $[0, 1]$ as follows. If a mutation is d positions away from the nearest element, $0 \leq d \leq w$, then the score s is given as:

$$s = \begin{cases} \frac{1}{d+1}, & d \leq w. \\ 0, & \text{otherwise}. \end{cases} \tag{2}$$

To identify the optimum setting for the window, w, we ran LOCO CV using values from $w = 1$ to $w = 10^6$ and found that $w = 10$ yielded the best performance (*Distance*, Table 3).

3.5 Kernel performance

We evaluated each of these kernels using LOCO-CV (Table 3,4). Conservation kernels yield relatively high accuracy for both non-coding and coding examples, consistent with previous studies showing the importance of conservation in non-coding regions (Kircher *et al.*, 2014; Shihab *et al.*, 2015). The spectrum kernel also scored well for both coding and non-coding regions, suggesting that it may learn patterns of regulatory motifs gained or lost through mutation. Genomic context features also appear amongst the top-performing kernels.

3.5.1 Kernel performance for non-coding variants

Features	Accuracy	Features	Accuracy
100-way cons.	0.91	GappedPeak	0.56
46-way cons.	0.91	BroadHMM	0.55
Distance	0.91	Histone Chip-Seq	0.55
Long RNA-Seq	0.78	Segmentation	0.55
RnaChip	0.72	RIP-Chip GeneST	0.54
Spectrum	0.71	TFBS Peak-Seq	0.53
RNA	0.66	Chia PET	0.53
Riken CAGE	0.68	DNase Uniform	0.52
Repeats	0.61	FDR peaks	0.52
NarrowPeak	0.60	TFBS Uniform	0.52
BroadPeak	0.59	FAIRE	0.51
Mapability	0.58	Footprints	0.51
GC content	0.57	SPP	0.51
Chromatin HMM (15)	0.56	RipSeq	0.50
Chromatin HMM (18)	0.56	Tiling	0.50
Short RNA-Seq	0.56		

Table 3. **Accuracy of individual feature groups in non-coding regions** shows that the conservation groups and distance to genomic features yield by far the highest accuracy of any group. Groups are sorted in descending order by balanced accuracy in LOCO-CV.

For non-coding regions, conservation scores and gene element distance features yielded by far the highest accuracy. Gene expression estimates given by RNA-Seq also provided good discrimination, as did evidence for RNA binding proteins and the nucleotide spectrum kernel. The final model also leverages evidence for chromatin interactions via the Chia PET assay, which is not the most accurate of the chromatin assays on its own, but improves accuracy in the context of these other groups (Section 3.6).

3.5.2 Kernel performance for coding variants

Features	Accuracy	Features	Accuracy
100-way cons.	0.84	Segmentation	0.53
46-way cons.	0.83	RIP-Chip GeneST	0.53
VEP features	0.75	TFBS Uniform	0.52
RNA	0.62	Chia PET	0.52
Long RNA-Seq	0.59	Mapability	0.52
Spectrum	0.59	TFBS Peak-Seq	0.52
RnaChip	0.58	DNase Uniform	0.51
BroadPeak	0.57	FAIRE	0.51
NarrowPeak	0.57	FDR peaks	0.51
GappedPeak	0.56	GC content	0.51
BroadHMM	0.56	SPP (TFBS)	0.51
Chromatin HMM (15)	0.56	Footprints	0.50
Chromatin HMM (18)	0.56	Repeats	0.50
Histone Chip-Seq	0.55	RipSeq	0.50
Riken CAGE	0.55	Tiling	0.50
Short RNA-Seq	0.54		

Table 4. **Accuracy of individual feature groups in coding regions** shows that the conservation scores and VEP features far outperform other feature groups. Groups are sorted in descending order by balanced accuracy in LOCO-CV.

In coding regions, conservation scores again yielded the highest accuracy, followed by VEP features (amino acid changes and consequence), gene expression estimates and RNA interaction features. Again the nucleotide spectrum kernel is among the top-performing groups. Interestingly, the final model improved when we added the *Segmentation* features that provide gene element information similar to the VEP *consequence* features (Section 3.6).

3.6 Data-level integration

The simplest kernel method for integrating different data sources is to combine the features from all sources into a single kernel. This allows a model to discriminate between classes by learning how features from one source may interact with features from other sources. Given more than 30 possible data sources, there are billions of possible combinations of feature groups, making exhaustive testing impractical. Instead, we used an approach similar to previous work in which we found that sequential learning could be an effective means to identify an optimal combination of groups (Rogers *et al.*, 2015). In this work, we use a forward selection method, in which we try all combinations of the top-performing kernel with the remaining kernels to identify the best two-kernel model. Next, we try all combinations of this two-kernel model with remaining kernels, and continue the process until accuracy plateaus or starts to decline (Figure 3).

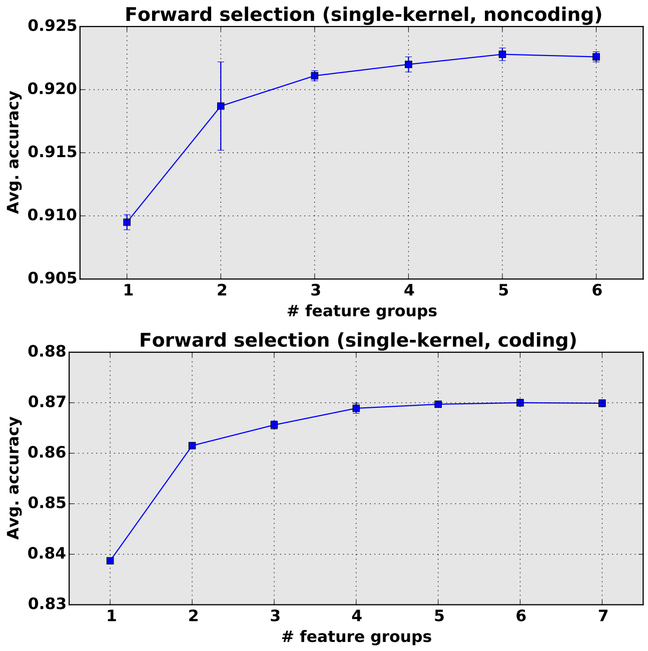

Fig. 3. Data-level integration for non-coding examples and coding examples: for data-level sequential learning we used balanced sets of 2,000 examples in LOCO-CV for each combination of features, and average accuracy over 10 LOCO-CV runs. **Top:** For non-coding regions, accuracy peaks at five feature groups (92.3% accuracy) after which it declines slightly. **Bottom:** For coding regions, accuracy reaches a nominal peak at six feature groups (87.0% accuracy).

For non-coding regions, the best model incorporated five feature groups (Figure 3, top): starting at 91.0% accuracy for the first group (*100-way conservation*), we see rapid improvement up to 92.1% accuracy for three groups, after which performance plateaus, reaching 92.3% accuracy with five groups. From that point onward, additional feature groups provide no evident advantage, as average accuracy declines. Hence our final non-coding region model uses a single kernel constructed from five feature groups.

For coding regions, our best model included six data sets (Figure 3, bottom): accuracy for the best feature group (again, *100-way conservation*) is 83.9% and climbs rapidly to 86.9% with four feature groups, reaching its peak with six feature groups at 87.0% accuracy. Our final coding-region model thus consists of a single kernel constructed from six feature groups.

3.7 Kernel-level integration

In previous studies we found that integration at the kernel level, using multiple kernel learning (MKL) and sequential learning strategies, tended to outperform integration at the data level (Rogers *et al.*, 2015; Shihab *et al.*, 2015, 2017b). We applied our sequential learning pipeline (Rogers *et al.*, 2015) to examples in both coding and non-coding regions. For non-coding regions, the best MKL model achieved 89% accuracy; for coding examples, the best MKL model achieved 85% accuracy. Both models thus yielded accuracy slightly below the best models from data-level integration.

3.7.1 Alternative classification models

For both coding and non-coding classifiers we investigated a variety of kernel-based models using the scikit-learn package (version 0.17.1) (Pedregosa *et al.*, 2011). We selected the package for its relatively robust performance and for the variety of models available. We evaluated seven different classification models to select the one yielding the best performance, and to observe changes in accuracy that could reflect potential overfitting (see Table 5). For each classifier we first used LOCO-CV to establish optimal parameters, then compared the accuracy, averaged over 10 runs, to identify the strongest performers in non-coding and coding regions. For each LOCO fold we used balanced training sets of 2,000 examples. In non-coding regions we found

that gradient boosting (Schapire, 2003), random forest (Ho, 1995) extra trees (Geurts *et al.*, 2006) and Adaboost (Freund and Schapire, 1995) yield the highest accuracy, with no significant differences observed between them. In coding regions, the rankings are nearly the same, the lone exception being the Naive Bayes classifier. Based on these results we selected gradient boosting for our models in both regions.

Algorithm	Non-coding	Coding
Gradient boosting	0.923	0.870
Extra trees	0.922	0.857
Random forest	0.921	0.854
Adaboost	0.918	0.855
Naive Bayes	0.912	0.562
SVM	0.908	0.833
Decision tree	0.880	0.801

Table 5. **Evaluation of different classification models** shows that Gradient Boosting yields the best performance in both non-coding and coding regions. Models were trained in LOCO-CV using balanced training sets of 2,000 examples. Average accuracy was computed over 10 runs. Models are sorted in order of decreasing accuracy on non-coding data.

4 Cautious classification

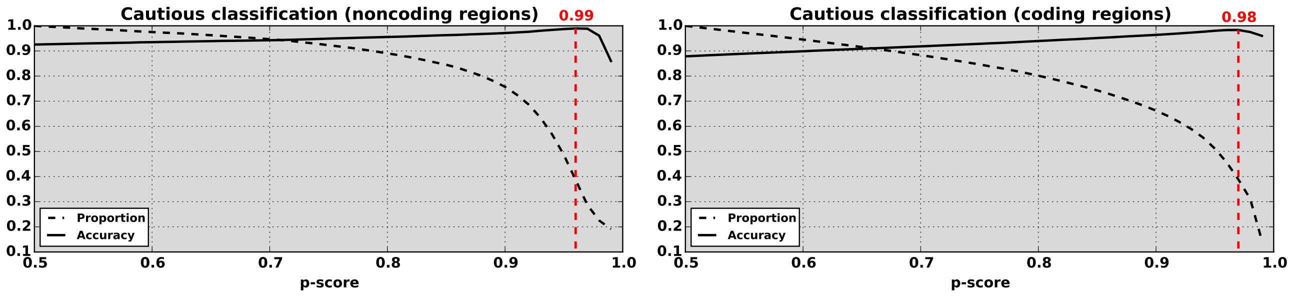

Fig. 4. Cautious classification curves show that prediction accuracy consistently improves with increasing p-scores up to the optimum thresholds. Left: accuracy increases monotonically for the non-coding model, reaching 99% at the optimum threshold $\tau = 0.96$, with predictions for nearly 40% of examples. **Right:** for the coding model, accuracy again increases monotonically, peaking at 98% at $\tau = 0.97$, with predictions for nearly 40% of examples.

By associating a confidence measure with each prediction, we can evaluate test accuracy against possible confidence measure thresholds, τ. Figure 4 shows model accuracy as a function of p-score cutoff for the non-coding and coding predictors in LOCO-CV, along with the proportion of examples whose p-scores exceed the cutoff. For the non-coding model (Figure 4, top), accuracy increases monotonically, reaching 99% at the optimum threshold $\tau = 0.96$. At this threshold, the model yields predictions for nearly 40% of examples. At lower thresholds, accuracy remains high while the model yields predictions for the vast majority of examples: at $\tau = 0.90$, the model yields predictions for more than 75% of examples, maintaining accuracy over 97%; at $\tau = 0.80$, it yields predictions for almost 90% of examples, with accuracy above 95%.

For the coding model (Figure 4, bottom) accuracy again increases monotonically, reaching 98% at its optimum threshold $\tau = 0.97$, yielding predictions for nearly 40% of examples. Once again we see that at slightly lower thresholds the model still achieves high accuracy while generating predictions for a much greater proportion of examples: at $\tau = 0.90$, it yields predictions for more than 66% of examples, with accuracy over 96%, and at $\tau = 0.80$, the coding model yields predictions for more than 80% of examples at 94.0%.

5 Results

To evaluate our models, we compared them with several state-of-the-art genome-wide predictors: CADD (Kircher *et al.*, 2014), DANN (Quang *et al.*, 2014), FATHMM-MKL (Shihab *et al.*, 2015) and GAVIN-adjusted CADD (van der Velde *et al.*, 2017). First we compared each model's test results from LOCO cross-validation with those of top competitors. Next, we also compared both models with top competitors on an independent set of ClinVar examples (April, 2017) (Landrum *et al.*, 2014). ClinVar uses the terms *benign, likely benign, likely pathogenic* and *pathogenic* to annotate each example. We selected *benign* and *likely benign* examples as negatives and *pathogenic* and *likely pathogenic* as positives. Some examples have both designations, so we removed these from our set. We also removed any examples that appeared in our training data. The final test sets thus contained 31,099 examples in non-coding regions (3,806 positive and 27,293 negative) and 62,884 examples in coding regions (26,023 positive and 36,861 negative).

5.1 Statistics definitions

In this work we use several statistics to evaluate classifier performance, including balanced accuracy, sensitivity, specificity, Matthews correlation coefficient (MCC) and positive predictive value (PPV). For any given test set, we count the number of true positives (TP), true negatives (TN), false positives (FP) and false negatives (FN), where pathogenic SNVs are considered "positive" and neutral SNVs are considered "negative". Hence pathogenic SNVs with p-scores $p > 0.5$ are counted as TP, whilst those with $p \leq 0.5$ are counted as FN. Similarly, neutral SNVs with p-scores $p \leq 0.5$ are counted as TN, whilst those with $p > 0.5$ are counted as FP. The statistics are then computed as follows:

$$\text{sensitivity} = \frac{TP}{TP + FN}$$

$$\text{specificity} = \frac{TN}{TN + FP}$$

$$\text{balanced accuracy} = 0.5 * (\text{sensitivity + specificity})$$

$$\text{PPV} = \frac{TP}{TP + FP}$$

$$\text{MCC} = \frac{TP * TN - FP * FN}{\sqrt{(TP + FP)(TP + FN)(TN + FP)(TN + FN)}}$$

In addition to these statistics, we also measure the area under the ROC curve (AUC). Note that we are unable to produce ROC curves for GAVIN, as it yields only predicted labels (*Pathogenic* or *Benign*). In addition, its adjusted scores are gene-specific and hence ranking comparisons cannot easily be made between scores from different genomic locations.

5.2 Non-coding variants

File	Acc.	AUC	Sens.	Spec .	MCC	PPV
FATHMM-XF	**0.93**	**0.97**	0.89	**0.97**	**0.86**	**0.91**
cautious (39% coverage)	**0.99**	**0.99**	**0.99**	**0.99**	**0.98**	**0.99**
FATHMM-MKL	0.90	0.95	0.85	0.94	0.80	0.87
GAVIN	0.83	—	0.68	**0.97**	0.71	**0.90**
CADD (v1.3)	0.71	0.93	0.94	0.49	0.41	0.44
DANN	0.67	0.91	**0.96**	0.38	0.35	0.40

Table 6. **FATHMM-XF outperforms all competitors on LOCO CV test examples for non-coding regions.** The new model yields higher accuracy, AUC, specificity and Matthews correlation (MCC) than its competitors when evaluated using LOCO-CV. At the optimum cautious classification threshold ($\tau = 0.96$), FATHMM-XF yields nearly perfect classification whilst yielding predictions for 38.7% of examples.

We compared the non-coding model test results from LOCO cross-validation with those of top competitors CADD, DANN, FATHMM-MKL and GAVIN-adjusted CADD. At the default threshold ($\tau = 0.5$), the new model outperforms all other methods, reaching 93% accuracy and 0.97 AUC (Table 6). In addition, the new model yields substantially higher MCC and PPV scores. At the cautious classification threshold ($\tau = 0.96$), the new model yields nearly perfect classification whilst yielding predictions for 38.7% of examples.

File	Acc.	AUC	Sens.	Spec.	MCC	PPV
FATHMM-XF	**0.89**	**0.97**	0.95	0.84	0.53	0.36
cautious (30% coverage))	**0.96**	**0.99**	**0.99**	**0.93**	**0.87**	**0.82**
FATHMM-MKL	0.88	0.95	0.94	0.82	0.49	0.33
GAVIN	0.87	—	0.82	**0.93**	**0.61**	**0.52**
CADD (v1.3)	0.64	0.95	0.98	0.30	0.18	0.12
DANN	0.61	0.95	**0.99**	0.23	0.15	0.11

Table 7. **FATHMM-XF outperforms competitors on unseen ClinVar examples for non-coding regions.** At the default threshold, the new model yields slightly higher statistics than its nearest competitor, FATHMM-MKL. However, we note that GAVIN yields higher Specificity, MCC and PPV values, as it yields the fewest false-positives on this benchmark set. At the cautious classification threshold, FATHMM-XF yields nearly perfect classification, achieving by far the highest scores, whilst yielding predictions for more than 30% of examples.

Testing all methods on the ClinVar non-coding data set, at the default threshold ($\tau = 0.5$), the new model matches or outperforms all other methods, reaching 89% accuracy and 0.97 area under the ROC curve (AUC, Table 7). FATHMM-MKL yields accuracy nearly as high, but overall it tends to under-perform the new model slightly. GAVIN also yields competitive accuracy, but achieves better MCC and PPV scores. At the cautious classification threshold ($\tau = 0.96$), the new model yields nearly perfect classification whilst yielding predictions for 30.9% of examples.

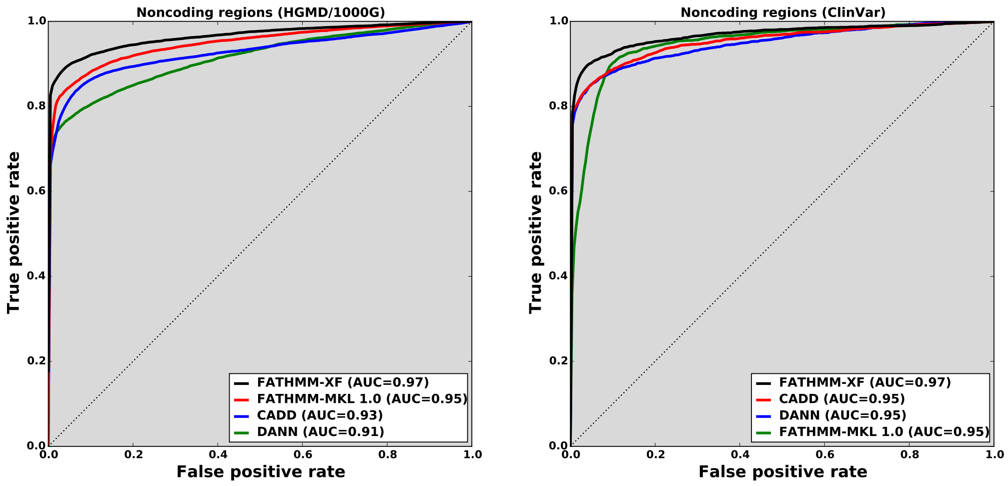

Fig. 5. ROC curves demonstrate that FATHMM-XF outperforms competitors in non-coding regions both on LOCO-CV test examples (left) and on the independent ClinVar test set (right).

File	Acc.	AUC	Sens.	Spec.	MCC	PPV
FATHMM-XF	**0.88**	**0.95**	0.89	**0.88**	0.74	0.77
cautious (39% coverage)	**0.98**	**0.99**	0.98	**0.99**	**0.96**	**0.97**
GAVIN	**0.89**	—	0.90	**0.88**	**0.76**	**0.80**
FATHMM-MKL	0.80	0.90	0.91	0.69	0.58	0.61
CADD (v1.3)	0.63	0.92	0.98	0.28	0.31	0.42
DANN	0.60	0.90	**0.99**	0.20	0.27	0.40

Table 8. **FATHMM-XF performs as well as any competitors on LOCO CV test examples in coding regions.** At the cautious classification threshold ($\tau = 0.97$) it yields significantly better performance, reaching 98% prediction accuracy for 38.8% of examples.

5.3 Coding variants

We compared the coding model test results from LOCO cross-validation with the same set of top competitors (Table 8). At the default threshold, FATHMM-XF outperforms all methods except GAVIN, whose accuracy at 89% is slightly higher than FATHMM-XF (88%). We note that GAVIN yields dramatically higher accuracy than the CADD predictions it is based upon; a similar approach applied to FATHMM-MKL or FATHMM-XF scores may yield similarly impressive results. At the cautious classification threshold ($\tau = 0.96$), FATHMM-XF again yields nearly perfect classification whilst yielding predictions for 39.1% of examples.

File	Acc.	AUC	Sens.	Spec.	MCC	PPV
FATHMM-XF	**0.88**	**0.96**	0.84	**0.92**	**0.76**	**0.83**
cautious (40% coverage)	**0.97**	**0.99**	0.94	**1.00**	**0.96**	**0.99**
GAVIN	**0.89**	—	0.90	0.87	0.74	0.76
FATHMM-MKL	0.80	0.90	0.91	0.70	0.56	0.58
CADD (v1.3)	0.63	0.91	0.98	0.29	0.30	0.38
DANN	0.60	0.89	**0.99**	0.20	0.25	0.36

Table 9. **FATHMM-XF outperforms most competitors on unseen ClinVar examples in coding regions.** At the default threshold ($\tau = 0.5$) the new model yields substantially higher accuracy than any competitors except GAVIN, which is nominally more accurate on this benchmark set. However, FATHMM-XF's AUC, Specificity, MCC and PPV scores are all higher than any competitor. At the cautious classification threshold, FATHMM-XF again achieves exceptionally high scores, whilst yielding predictions for more than 40% of examples.

Testing all methods on the ClinVar coding data set, at the default threshold FATHMM-XF matches or outperforms nearly all methods, reaching 88% accuracy and 0.96 AUC (Table 9). Again we see that GAVIN yields nominally higher accuracy, but with reduced Specificity, MCC and PPV. GAVIN also yields competitive accuracy, but achieves better MCC and PPV scores. At the cautious classification threshold ($\tau = 0.96$), FATHMM-XF again yields exceptional performance whilst yielding predictions for 42.4% of examples.

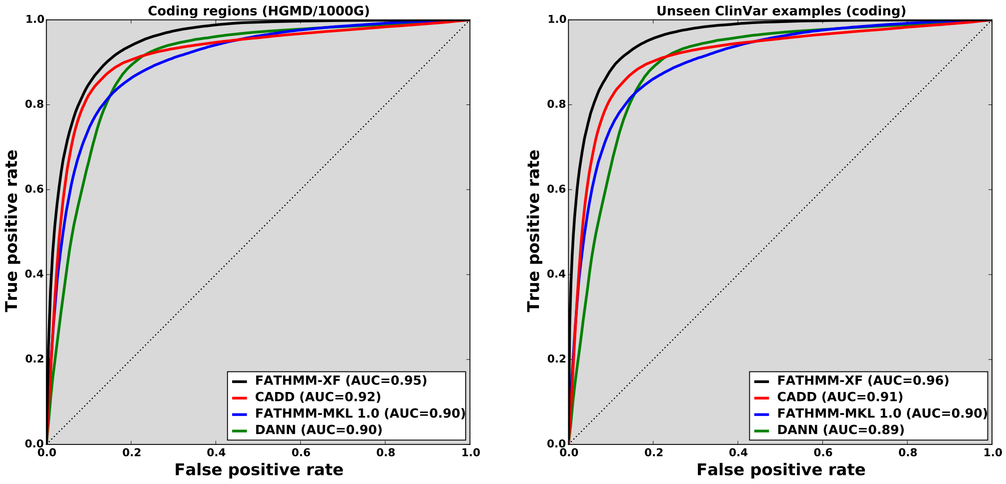

Fig. 6. ROC curves demonstrate that FATHMM-XF outperforms all competitors in coding regions both on LOCO cross-validation test examples (left) and on the independent ClinVar test set (right).

6 Optimum performance

GAVIN provides unequivocal *Pathogenic* (positive) and *Benign* (negative) predictions for all examples, but the other methods provide scores, along with default thresholds for identifying positive and negative predictions. To explore further the performance of the ranking methods, we iterated over all scores for each and evaluated the balanced accuracy and false positive rates (FPR) on our training data and on the benchmark test sets (Supplementary Figures 7 through 10). This provides a comprehensive view of their performance, and potentially may lead to alternative thresholds we can use to present each of them in its best possible light. Overall we found that *FATHMM-XF* and *FATHMM-MKL* yield relatively consistent performance across both training and test data sets, except at the extreme ends of their score ranges, whilst CADD and DANN are far more sensitive to the threshold being used.

6.1 Non-coding regions

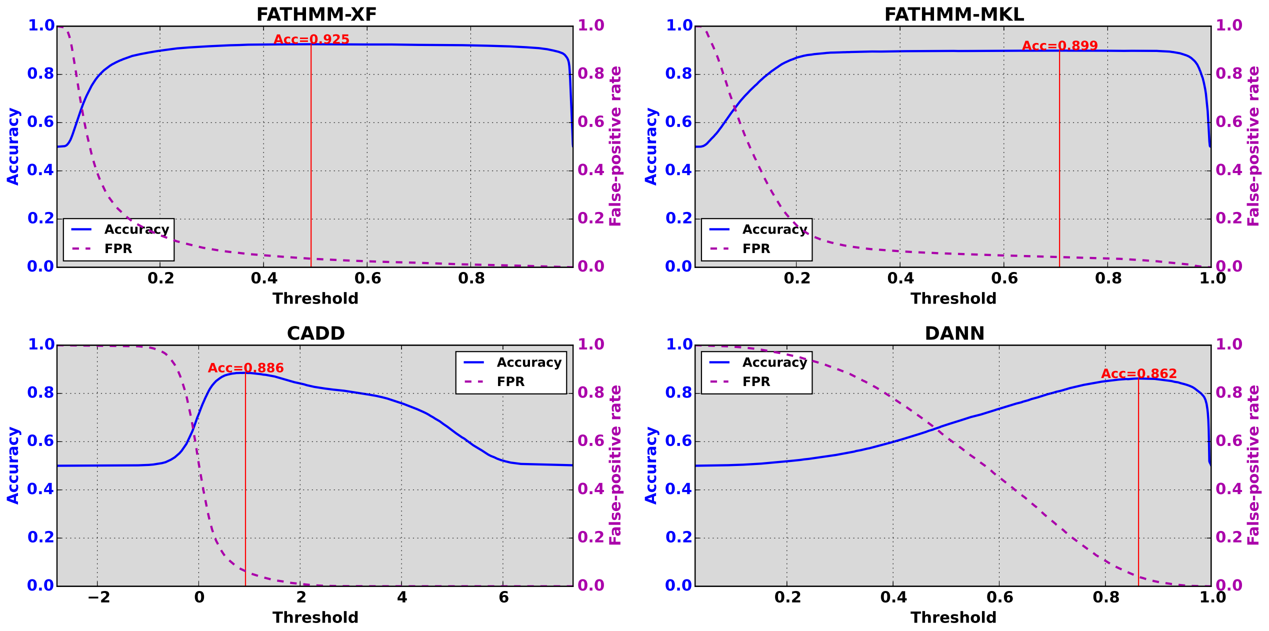

Fig. 7. Balanced accuracy as a function of threshold on FATHMM-XF training data for non-coding regions shows that FATHMM-XF and FATHMM-MKL yield highly consistent performance while CADD and DANN are more sensitive to thresholds. For FATHMM-XF, peak balanced accuracy of 92.5% is reached at a threshold of 0.492; FATHMM-MKL, reaches balanced accuracy of 89.9% at a threshold of 0.707; CADD reaches 88.6% at a threshold of 0.922, and DANN reaches 86.2% at 0.862. All methods except DANN achieve a relatively low false-positive rate (FPR) near the middle of their score ranges.

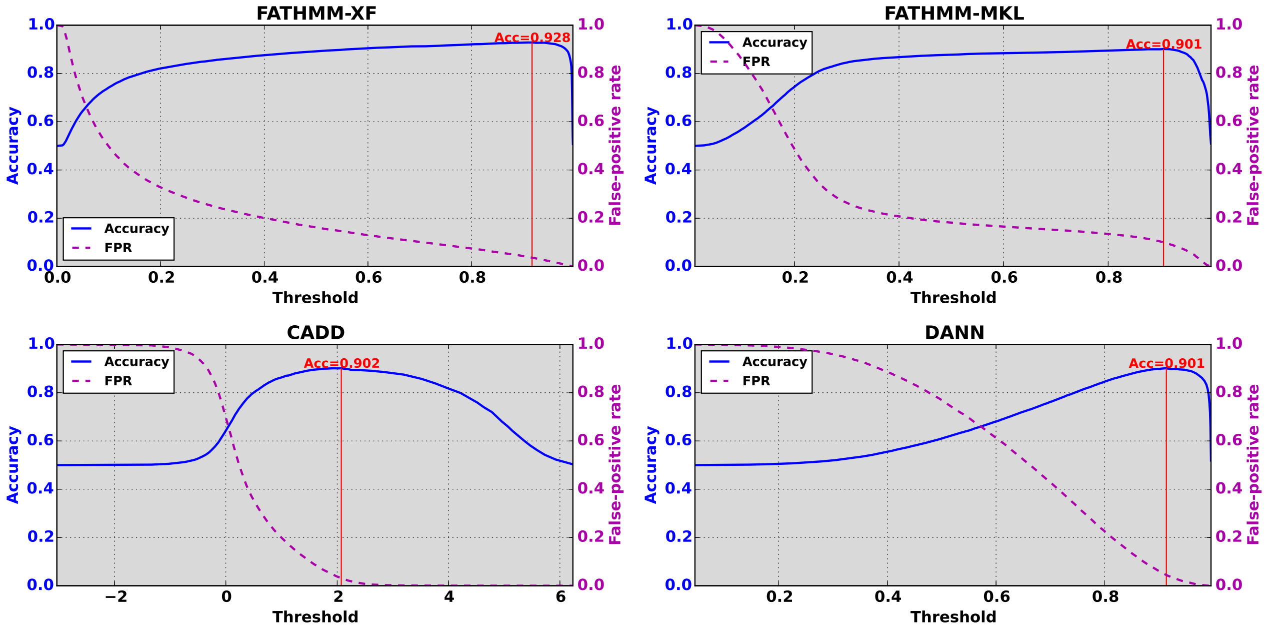

Fig. 8. Balanced accuracy as a function of threshold on the ClinVar benchmark for non-coding regions shows that FATHMM-XF and FATHMM-MKL yield consistent performance while CADD and DANN more sensitive to thresholds. To achieve peak accuracy, all methods require a higher threshold than on the FATHMM-XF training data: FATHMM-XF reaches 92.8% accuracy at a threshold of 0.917; FATHMM-MKL reaches 90.1% accuracy at a threshold of 0.906; CADD reaches 90.2% accuracy at a threshold of 2.07, and DANN reaches 90.1% accuracy at a threshold of 0.913. Of the four methods, CADD yields the lowest FPR near the top end of its scoring range, while DANN yields the highest FPR throughout most of its range.

6.2 Coding regions

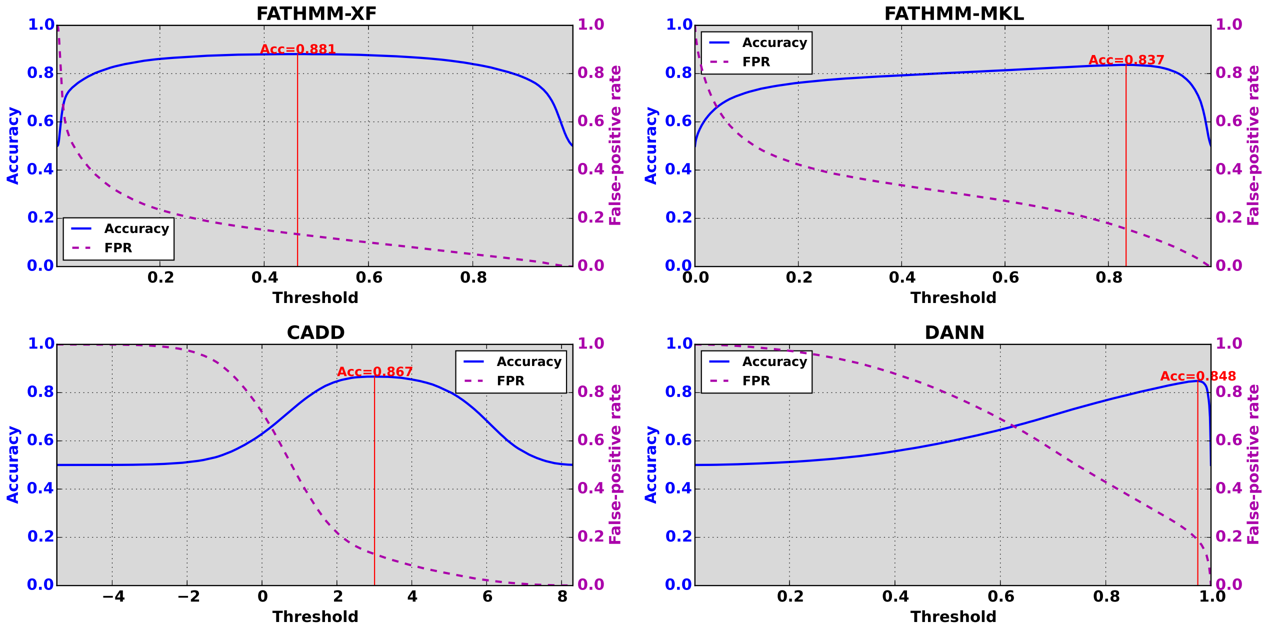

Fig. 9. Balanced accuracy as a function of threshold on FATHMM-XF training data for coding regions shows that FATHMM-XF and FATHMM-MKL yield highly consistent performance while CADD and DANN are more sensitive to thresholds. For FATHMM-XF, peak balanced accuracy of 88.1% is reached at a threshold of 0.464; FATHMM-MKL, reaches balanced accuracy of 83.7% at a threshold of 0.834; CADD reaches 86.7% at a threshold of 3.01, and DANN reaches 84.8% at 0.974. All methods except DANN achieve a relatively low false-positive rate (FPR) near the middle of their score ranges.

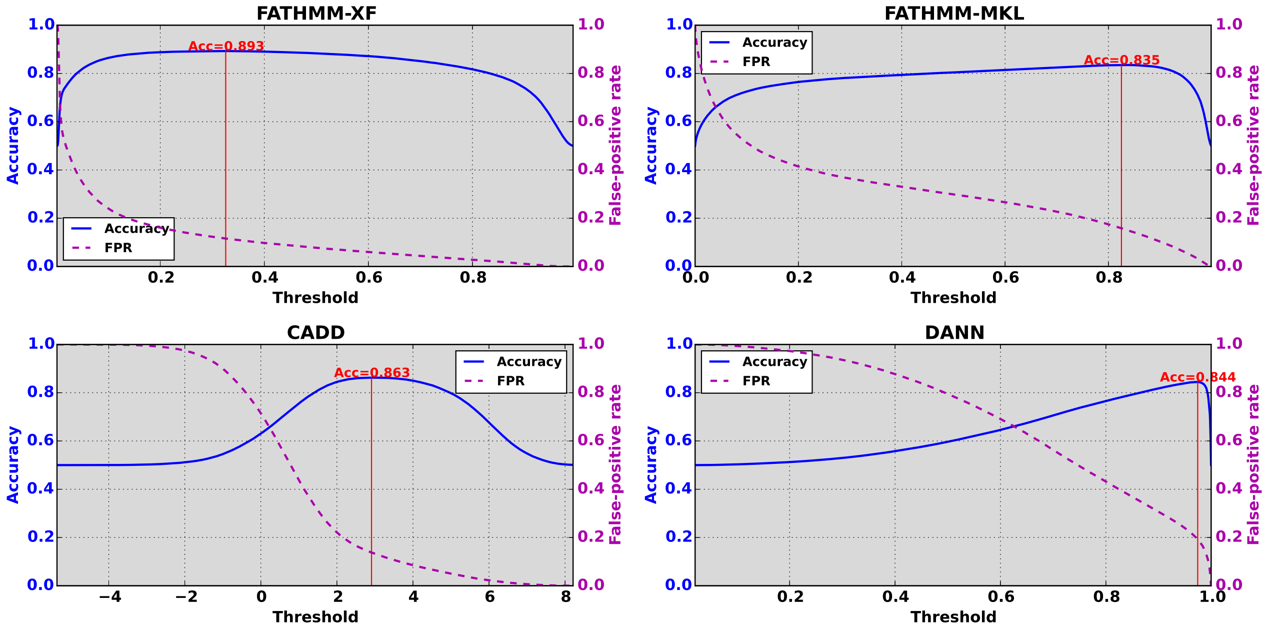

Fig. 10. Balanced accuracy as a function of threshold on the ClinVar benchmark for non-coding regions shows that FATHMM-XF and FATHMM-MKL yield consistent performance while CADD and DANN are sensitive to thresholds. To achieve peak accuracy, all methods require a higher threshold than on the FATHMM-XF training data: FATHMM-XF reaches 89.3% accuracy at a threshold of 0.326; FATHMM-MKL reaches 83.5% accuracy at a threshold of 0.825; CADD reaches 86.3% accuracy at a threshold of 2.91, and DANN reaches 84.4% accuracy at a threshold of 0.974. FATHMM-XF yields relatively low FPR for most of its scoring range, while DANN yields relatively high FPR throughout its range.

7 Website

Pre-computed predictions for the GRCh37/hg19 version of the human genome are available online at fathmm.biocompute.org.uk/fathmm-xf. Users query the database by entering comma-separated strings consisting of chromosome, position, reference and mutant alleles, or they may upload files in VCF format. The webserver can process queries at an approximate rate of $3,300$ non-coding queries per minute or $50,000$ coding queries per minute. The difference is due to the relative size of the two databases, as the non-coding database is by far the larger of the two, with nearly 8.5 billion predictions, compared with 100 million predictions in coding sequence. Users who submit very large lists of queries may provide an e-mail address where they will receive notification when the job has finished. The pre-computed databases are also available for download along with a Python script that performs the same queries as the website.

The full set of predictions are also available as tracks on the Genome Tolerance Browser (GTB) (Shihab *et al.*, 2017a). Users may access the tracks at gtb.biocompute.org.uk.

References

Adzhubei, I. *et al.* (2010). A method and server for predicting damaging missense mutations. *Nature Methods*, **7**, 248–249.

Bernstein, B. E. *et al.* (2010). The NIH roadmap epigenomics mapping consortium. *Nat. Biotechnology*, **28**(10), 1045–1048.

Blanchette, M., Kent, W. J., Riemer, C., Elnitski, L., Smit, A. F., Roskin, K. M., Baertsch, R., Rosenbloom, K., Clawson, H., Green, E. D., *et al.* (2004). Aligning multiple genomic sequences with the threaded blockset aligner. *Genome research*, **14**(4), 708–715.

Campbell, C. and Ying, Y. (2011). *Learning with Support Vector Machines*. Morgan and Claypool.

Charlesworth, B., Coyne, J., and Barton, N. (1987). The relative rates of evolution of sex chromosomes and autosomes. *The American Naturalist*, **130**(1), 113–146.

Freund, Y. and Schapire, R. E. (1995). A decision-theoretic generalization of on-line learning and an application to boosting. In *European conference on computational learning theory*, pages 23–37. Springer.

Geurts, P., Ernst, D., and Wehenkel, L. (2006). Extremely randomized trees. *Machine learning*, **63**(1), 3–42.

Ho, T. K. (1995). Random decision forests. In *Document Analysis and Recognition, 1995., Proceedings of the Third International Conference on*, volume 1, pages 278–282. IEEE.

Kircher, M. *et al.* (2014). A general framework for estimating the relative pathogenicity of human genetic variants. *Nat. genetics*, **46**(3), 310–315.

Kundaje, A., Meuleman, W., Ernst, J., Bilenky, M., Yen, A., Heravi-Moussavi, A., Kheradpour, P., Zhang, Z., Wang, J., Ziller, M. J., *et al.* (2015). Integrative analysis of 111 reference human epigenomes. *Nature*, **518**(7539), 317–330.

Landrum, M. J. *et al.* (2014). ClinVar: public archive of relationships among sequence variation and human phenotype. *Nucleic Acids Res.*, **42**(D1), D980–D985.

Leslie, C. S., Eskin, E., and Noble, W. S. (2002). The spectrum kernel: A string kernel for SVM protein classification. In *Pacific Symposium on Biocomputing*, volume 7, pages 566–575. World Scientific.

McLaren, W. *et al.* (2016). The ENSEMBL Variant Effect Predictor. *Genome Biology*, **17**(1), 122.

Pedregosa, F., Varoquaux, G., Gramfort, A., Michel, V., Thirion, B., Grisel, O., Blondel, M., Prettenhofer, P., Weiss, R., Dubourg, V., Vanderplas, J., Passos, A., Cournapeau, D., Brucher, M., Perrot, M., and Duchesnay, E. (2011). Scikit-learn: Machine learning in Python. *Journal of Machine Learning Research*, **12**, 2825–2830.

Pollard, K. S., Hubisz, M., Rosenbloom, K., and Siepel, A. (2010). Detection of non-neutral substitution rates on mammalian phylogenies. *Genome Research*, **20**, 110–121.

Quang, D. *et al.* (2014). DANN: a deep learning approach for annotating the pathogenicity of genetic variants. *Bioinformatics*, **31**, 761–763.

Ritchie, G., Dunham, I., Zeggini, E., and Flicek, P. (2014). Functional annotation of noncoding sequence variants. *Nature Methods*, **11**, 294–296.

Rogers, M., Campbell, C., Shihab, H., Gaunt, T., Mort, M., and Cooper, D. (2015). Sequential data selection for predicting the pathogenic effects of sequence variation. In *Bioinformatics and Biomedicine (BIBM), 2015 IEEE International Conference on*, pages 639–644.

Schapire, R. E. (2003). The boosting approach to machine learning: An overview. In *Nonlinear estimation and classification*, pages 149–171. Springer.

Shihab, H. *et al.* (2015). An integrative approach to predicting the functional effects of non-coding and coding sequence variation. *Bioinformatics*, **31**, 1536–1543.

Shihab, H. A., Rogers, M. F., Ferlaino, M., Campbell, C., and Gaunt, T. R. (2017a). GTB–an online genome tolerance browser. *BMC Bioinformatics*, **18**(1), 20.

Shihab, H. A., Rogers, M. F., Campbell, C., and Gaunt, T. R. (2017b). Hipred: an integrative approach to predicting haploinsufficient genes. *Bioinformatics*, **33**(12), 1751–1757.

Siepel, A., Bejerano, G., Pedersen, J., Hinrichs, A., Hou, M., Rosenbloom, K., Clawson, H., Spieth, J., Hillier, L., Richards, S., Weinstock, G., Wilson, R., Gibbs, R., Kent, W., Miller, W., and Haussler, D. (2005). Evolutionarily conserved elements in vertebrate, insect, worm, and yeast genomes. *Genome Research*, **15**, 1034–1050.

Stenson, P. D. *et al.* (2017). The human gene mutation database: towards a comprehensive repository of inherited mutation data for medical research, genetic diagnosis and next-generation sequencing studies. *Human Genetics*, **136**, 665–677.

The 1000 Genomes Project Consortium (2012). An integrated map of genetic variation from 1,092 human genomes. *Nature*, **491**, 56–65.

The ENCODE Project Consortium (2012). An integrated encyclopedia of DNA elements in the human genome. *Nature*, **489**, 57–74.

van der Velde, K. J. *et al.* (2017). Gavin: Gene-aware variant interpretation for medical sequencing. *Genome Biology*, **18**(1), 6.
